# Safety Evaluation of *Zingiber cassumunar* Roxb. Rhizome Extract: Acute and Chronic Toxicity Studies in Rats

**DOI:** 10.1155/2014/632608

**Published:** 2014-11-16

**Authors:** Sittichai Koontongkaew, Orapan Poachanukoon, Seewaboon Sireeratawong, Thaweephol Dechatiwongse Na Ayudhya, Parirat Khonsung, Kanjana Jaijoy, Ruedee Soawakontha, Monraudee Chanchai

**Affiliations:** ^1^Faculty of Dentistry, Thammasat University, Rangsit Campus, Paholyothin Road, Klongluang, Pathumthani 12121, Thailand; ^2^Medicinal Herb Research Unit for Asthma, Thammasat University, Phaholyothin Road, Klongluang, Pathumthani 12121, Thailand; ^3^Faculty of Medicine, Thammasat University, Paholyothin Road, Klongluang, Pathumthani 12120, Thailand; ^4^Faculty of Medicine, Chiangmai University, Chiangmai 50200, Thailand; ^5^Faculty of Pharmaceutical Science, Huachiew Chalermprakiet University, Bangna-Trad Road, Bang Phli, Samut Prakan 10540, Thailand; ^6^Faculty of Pharmacy, Mahidol University, Si Ayutthaya Road, Bangkok 10400, Thailand

## Abstract

*Zingiber cassumunar* Roxb. has been used for traditional medicine, but few studies have described its potential toxicity. In this study, the acute and chronic oral toxicity of *Z. cassumunar* extract granules were evaluated in Sprague-Dawley rats. The extract at a single dose of 5000 mg/kg body weight did not produce treatment related signs of toxicity or mortality in any of the animals tested during the 14-day observation period. However, a decrease in body weights was observed in treated males (*P* < 0.05). The weights of lung and kidney of treated females were increased (*P* < 0.05). Treated males were increased in spleen and epididymis weights (*P* < 0.05). In repeated dose 270-day oral toxicity study, the administration of the extracts at concentrations of 0.3, 3, 30, 11.25, 112.5, and 1,125 mg/kg body weight/day revealed no-treatment toxicity. Although certain endpoints among those monitored (i.e., organ weight, hematological parameters, and clinical chemistry) exhibited statistically significant effects, none was adverse. Gross and histological observations revealed no toxicity. Our findings suggest that the *Z. cassumunar* extract granules are well tolerated for both single and chronic administration. The oral no-observed-adverse-effect level (NOAEL) for the extract was 1,125 mg/kg body weight/day for males and females.

## 1. Introduction

*Zingiber cassumunar* Roxb. (Family* Zingiberaceae*) known locally as “Phlai” in Thai is a perennial herb, consisting of underground rhizomes. Traditionally, the rhizome of this plant has been used for treatment of inflammation, muscle and joint problems, menstrual disorders, abscesses, and skin diseases and wound healing [1]. Phytochemical investigations of* Z. cassumunar* rhizomes have revealed the presence of phenylbutanoids, cyclohexene derivatives, naphthoquinones, vanillin, vanillic acid, veratric acid [2–5], terpenoids, *β*-sitosterol, and curcuminoids [6–9].

**Figure 1:**
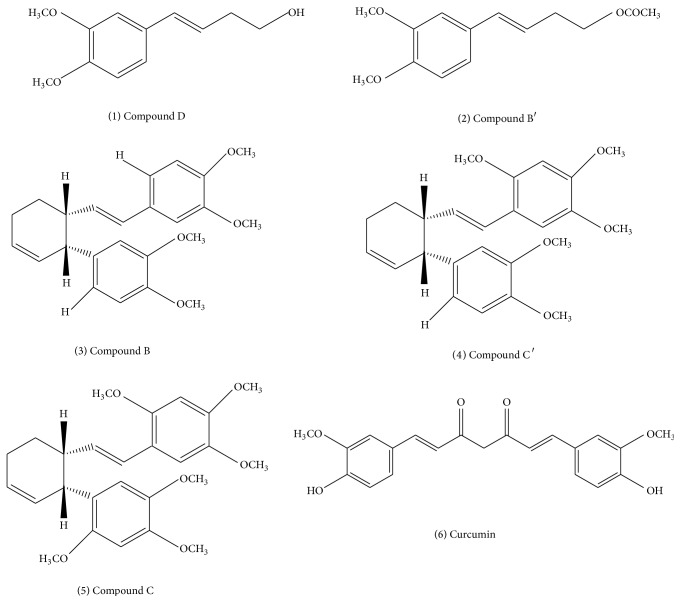
The structures of potential active compounds in* Zingiber cassumunar* Roxb.

In combination with other medicinal plants,* Z. cassumunar* was found to be effective in relieving asthmatic symptoms in children and adults [10, 11]. Several isolated compounds have been found to possess anti-inflammatory activity, for example, two phenylbutanoids, (*E*)-4-(3′, 4′-dimethoxyphenyl) but-3-en-1-ol; compound D (**1**) and (*E*)-4-(3′,4′-dimethoxyphenyl) but-3-en-1-yl acetate; compound B′ (**2**); three cyclohexene derivatives, namely,* cis*-3-(3′,4′dimethoxyphenyl)-4-[(*E*)-3′′′,4′′′-dimethoxystyryl] cyclohex-1-ene; compound B (**3**),* cis*-3-(3′,4′-dimethoxyphenyl)-4-[(*E*)-2′′′,4′′′,5′′′-trimethoxystyryl] cyclohex-1-ene; compound C′ (**4**) and* cis*-3-(2′,4′,5′-trimethoxyphenyl)-4-[(*E*)-2′′′,4′′′,5′′′-trimethoxystyryl] cyclohex-1-ene; compound C (**5**) and curcumin (**6**) [12–16]. The chemical structures of these compounds are shown in Figure 1. Moreover, smooth muscle relaxant, uterine relaxant, and antihistaminic activity of compound D [17, 18] and antioxidant activity of curcumin [16] have been reported.

Despite the centenary use of this plant, there are considerably few published toxicity studies on its toxicity. Acute and chronic toxicity studies of the hexane extracts of* Z. cassumunar* were performed in rats, and no toxic effects were found [19]. However, toxicity assessment in rats and monkeys demonstrated that the plant might be toxic to the liver [20]. Considering previous toxicity assessments of* Z. cassumunar*, it is not clear whether it produces any significant toxicity. In addition, there is limited or no documented toxicological studies, which can be used to ascertain the safety index of the herbal preparation. The present study therefore aims at investigating acute and chronic oral toxicity of* Z. cassumunar* extracts applying the recommended Organization of Economic Cooperation and Development (OECD) and World Health Organization (WHO) guidelines for safety or dose-dependent toxicity in rats.

## 2. Materials and Methods

### 2.1. Plant Material and Chemicals

Fresh rhizomes of* Z. cassumunar* were purchased from a medicinal herb supplier in Chiangmai Province and identified as* Z. cassumunar* at the Medicinal Plant Research Institute, Department of Medical Sciences, Ministry of Public Health, Nonthaburi, Thailand. The voucher specimen has been deposited at the Department of Pharmacognosy, Faculty of Pharmacy, Mahidol University. All solvents used in the present study were analytical grade and mostly purchased from Merck KGaA (Darmstadt, Germany). All other chemicals were obtained from Sigma-Aldrich (St. Louis, MO).

### 2.2. Marker Compounds

The isolation and structure identification of marker compounds** 1**–**5** from* Z. cassumunar* Roxb. rhizomes were carried out by the methods previously described [2, 3, 6]. They have been kept as references at Oral Biology Unit, Faculty of Dentistry, Thammasat University. Curcumin (**6**) was obtained from Sigma-Aldrich.

### 2.3. Preparation and Identification of Tested Substances

The fresh rhizomes of* Z. cassumunar* were sliced and the essential oil was removed by steam distillation. The plant residue was dried at 60°C and then macerated twice with ethanol. After filtration, the filtrate was concentrated with a rotary evaporator, giving the ethanolic* Z. cassumunar* extract, with a yield of 9.1% (drug extract ratio, DER = 11 : 1). The resulting* Z. cassumunar* extracts were used for granular preparation.

A wet granulation method was used to prepare* Z. cassumunar* extract granules. Briefly, the ethanolic extract of* Z. cassumunar* was mixed with suitable excipients to provide granulated substance. The excipients were dibasic calcium phosphate, microcrystalline cellulose, cornstarch, sodium starch glycolate, and polyvinylpyrrolidone K 30 (PVPK30). The concentration of* Z. cassumunar* extract in the granulated substance was 20% w/w. All steps of granular preparation were carried out at Faculty of Pharmacy, Mahidol University, Bangkok, Thailand.

In order to ensure the presence of potential active constituents,* Z. cassumunar* extract granules were subjected to thin layer chromatography (TLC) for detection of marker compounds** 1**–**6**, according to Thai Herbal Pharmacopoeia [21, 22]. Briefly,* Z. cassumunar* extract granules were dissolved in ethanol. The dissolved granules along with marker compounds were subjected to TLC for the comparison of hR_f_ values. The chromatographic analysis was carried out using a precoated silica gel 60 GF 254 plate (Merck). A mixture of hexane/ethyl acetate (70 : 30, v/v) was used as a mobile phase. To visualize the resolved compounds, the TLC plates were exposed to fluorescent indicator ultraviolet ray (*λ* = 254 nm) and then sprayed with* p*-anisaldehyde/sulfuric acid reagents, followed by heating at 110°C for 10 min. The respective hR_f_ values of certain bioactive substances in the* Z. cassumunar* extract granules were calculated and compared with that of the marker compounds.

### 2.4. Experimental Animals

Both female and male Sprague-Dawley rats that weighted 200–280 g and aged seven weeks were obtained from the National Laboratory Animal Center, Nakorn Pathom, Thailand. Animals were first left for 7 days to acclimatize to laboratory conditions. They were maintained at 25 ± 1°C under a light/dark cycle of 12 h. Animals were randomly allocated to control and treatment groups (10 rats/sex/group). The experimental protocol was approved by The Animal Ethics Committee, Thammasat University, Pathumthani, Thailand.

### 2.5. Acute Toxicity Assessment

An acute toxicity test was performed according to WHO guideline [23] and OECD guideline for acute oral toxicity [24]. Rats were deprived of food except water for 16−18 h prior to dosing on day 0. They were assigned to four groups of 10 animals each.* Z. cassumunar* extract granules were administered orally (only once) at a single dose of 5,000 mg/kg body weight (bw) to experimental groups (10 males and 10 females). The control groups (10 males and 10 females) received placebo granules. Neither food nor drinking water was given up to 4 h after treatments. The general behavior of the rats was continuously monitored for 1 h after dosing, periodically during the first 24 h, with special attention given during the first 4 h and once daily further for a period of 14 days. The rats were weighed and visual observations for mortality, behavioral patterns (salivation, fur, lethargy, and sleep), physical appearance, injury, pain, and signs of illness were conducted once daily during the period.

At the end of the experiment, all surviving animals were euthanized through intraperitoneal injection of pentobarbital sodium (100 mg/kg). Selected organs, including lungs, heart, livers, kidneys, spleens, pancreas, adrenals, uterus, ovaries, testes, epididymis, and brain were excised and weighed. The gross pathological features of the organs were observed.

### 2.6. Chronic Toxicity Assessment

Chronic toxicity study of* Z. cassumunar* extract granules was assessed according to WHO and OECD guidelines [23, 25]. Healthy rats of both sexes were randomly assigned into one of seven groups: a control group and six treatment groups (*n* = 20; 10 males and 10 females).* Z. cassumunar* extract granules were administered orally on daily basis for 270 days at the doses of 0.3, 3, 30, 11.25, 112.5, and 1,125 mg/kg bw while the control group received placebo granules. Two additional groups were devised as the satellite groups in order to observe the reverse sign of any toxicity. The two satellite groups were orally administered with the extracts at a daily dose of 30 or 1,125 mg/kg bw for 270 days, and there was no further treatment for the following 28 days before termination of the study. The rats were weighed and visual observations for mortality, behavioral pattern, physical appearances, injury, pain, and signs of illness were conducted once daily during that period. Surviving rats were terminated on day 270 in the main toxicity study and on day 298 in the recovery study. The animals were fasted for 16–18 h prior to sample collection. They were anesthetized through intraperitoneal injection of pentobarbital sodium. Blood samples were collected via cardiac puncture into nonheparinized and EDTA-containing tubes for biochemical and hematological analyses, respectively. The selected organs were excised, weighed, and examined macroscopically. The organs were preserved in 10% buffered formalin for histopathological examinations.

### 2.7. Hematological and Blood Chemical Analyses

Heparinized blood (2.5 mL) was subjected to hematological analysis using an automatic analyzer K-1000 (Sysmex; Kobe, Japan). The following parameters were determined: red blood cell count (RBC), white blood cell count (WBC), hemoglobin concentration (HGB), the hematocrit (HCT), mean corpuscular volume (MCV), mean corpuscular hemoglobin (MCH), mean corpuscular hemoglobin concentration (MCHC), platelet count (PLT), and the percentage of differentiated white blood cells including neutrophil (NEUT), lymphocyte (LYMPH), monocyte (MONO), eosinophil (EO), and basophil (BASO).

For clinical chemistry determination, serum was prepared from nonheparinized blood (5 mL). Glucose (GLU), blood urea nitrogen (BUN), creatinine (CRE), total protein (TP), albumin (ALB), total bilirubin (T-BIL), direct bilirubin (D-BIL), serum glutamic-oxaloacetic transaminase (SGOT), serum glutamine-pyruvic transaminase (SGPT), and alkaline phosphatase (ALP) were measured. These parameters were determined by the COBAS INTEGRA system (Roche Diagnostic System, Indianapolis, IN).

### 2.8. Pathological Examinations

Necropsy was performed on all animals including those which died during experiments. Macroscopic external features of certain organs including of lungs, heart, livers, kidneys, spleens, adrenals, uterus, ovaries, testes, epididymis, and brain were examined. The organs were fixed in 10% buffered formalin. Fixed samples were processed routinely and embedded in paraffin wax. Sections of 5 *μ*m thickness were cut, stained with hematoxylin and eosin (H&E), and examined under the light microscope by a pathologist. The microscopic features of the organs obtained from both sexes were compared with the corresponding organs of the controls.

### 2.9. Statistical Analyses

Data were expressed as mean ± standard error (SEM) unless otherwise stated. Statistical analyses were performed using SPSS v.22 (SPSS, Chicago, IL). The difference between experimental and control groups was separately evaluated for males and females. The homogeneity of variance and normality distribution were tested with Levene's and Kolmogorov-Smirnov tests, respectively. Independent Student's *t*-test was used to compare any significant difference between two groups. One way analysis of variance (ANOVA) followed by Dunnett's post hoc test was used for statistical comparison between control and various treated groups. *P* values of 0.05 or less were considered to be statistically significant.

## 3. Results

### 3.1. Preparation and Identification of Active Substances

**Figure 2:**
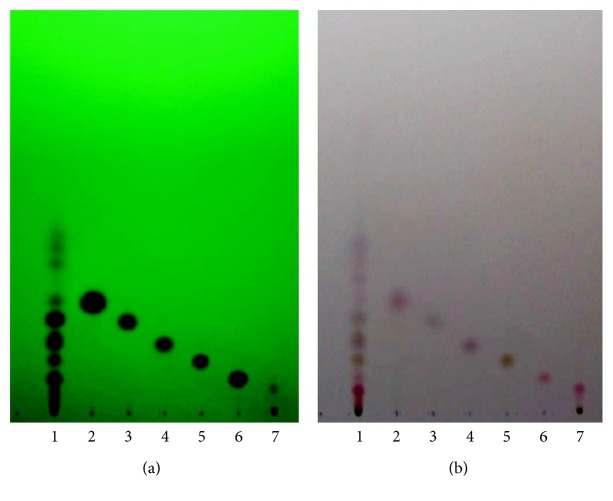
TLC analyses of* Z. cassumunar* extractgranules. Plate (a) was photographed under UV 254 nm light. Plate (b) was stained with *p*-anisaldehyde/sulfuric acid reagent and heated at 110°C for 10 min. Lanes 1–7 correspond to sample, compound B′, compound B, compound C′, compound C, compound D, and curcumin, respectively.

The TLC pattern of* Z. cassumunar* extract granules showed 13 prominent spots. Six spots at hR_f_ 13, 18, 25, 32, 44, and 51 corresponded to the spots of curcumin, compound D, compound C, compound C′, compound B, and compound B′, respectively (Figure 2). The separation achieved with TLC in the present study was consistent with that reported in Thai Herbal Pharmacopoeia [21].

### 3.2. Acute Toxicity Assessment

Lethal effects were not observed within 14 days after rats were fed with a single dose of* Z. cassumunar* extract granules (5000 mg/kg bw). No behavioral changes were observed during the observation period. However, the body weight of male rats treated with* Z. cassumunar* extract granules was significantly lower than that of the control group at day 14 (Table 1). In contrast, there was no significant difference in body weights between the control and treated females. Therefore, the LD_50_ value for oral administration of* Z. cassumunar* extract granules is greater than 5000 mg/kg bw.

Absolute organ weights of most internal organs of treated male and female rats were similar to their concurrent controls except for significant increase in the weights of lung and kidney of treated females and in spleen and epididymis weights of treated males (Table 2). However, the internal organs of treated animals showed no pathological abnormality. Therefore, our findings suggest that the* Z. cassumunar* extract granules used in the present study are considered to be orally nontoxic in rats.

After sacrifice on the 14th day, macroscopic and gross pathology observations conducted at the necropsy examination revealed no visible lesions in treated and control animals.

### 3.3. Chronic Toxicity Assessment

#### 3.3.1. Effects on Body and Organ Weights

In the chronic toxicity study,* Z. cassumunar* extract granules at doses of 0.3, 3, 30, 11.25, and 112.5 mg/kg bw/day, given orally for 270 days, did not produce clinical toxicity signs and death of rats during the experimental period. Compared with the control group, the body weight gain of treated rats was not significantly different. In addition, there was no significant difference in body weights between satellite and control groups (Table 3). Effects of* Z. cassumunar* administration on organ weights are presented in Table 4. Basically, there was no significant difference in absolute organ weights in most treated rats, compared to that of the controls. However, a decrease in kidney and spleen weights was observed in high-dose female rats (*P* < 0.05).

#### 3.3.2. Hematology

The hematological investigation demonstrated that red blood cell counts were statistically significant lower in female rats treated with* Z. cassumunar *extract granules at doses of 30 and 1,125 mg/kg bw/day for 270 and 298 days (*P* < 0.05) (Table 5). Hematocrit levels were significantly lower in the female rats given* Z. cassumunar *extract granules at 30 mg/kg bw/day for 298 days. However, the mean corpuscular hemoglobin of the treated rats was significantly higher compared to the control group (*P* < 0.05). No hematological change was observed in all male groups, except for a decrease in hemoglobin levels of the group given the* Z. cassumunar* extract granules at 30 mg/kg bw/day for 270 days.

After recovery from dosing, it was found that white blood cells were significantly lower in female satellite groups compared to the concurrent control. Neutrophil counts were statistically higher in female rats treated with* Z. cassumunar* extract granules at 3 mg/kg/bw/day for 270 days compared to the control. Lymphocytes were statistically decreased in all female rats treated with* Z. cassumunar* extract granules. In contrast, there was no obvious change in the number of white blood cells of experimental males except for the increase in neutrophil in male rats treated with* Z. cassumunar* extract granules at 0.3 mg/kg body weight for 270 days.

#### 3.3.3. Clinical Chemistries

Blood chemistry analyses showed significant increases in BUN in female rats treated with* Z. cassumunar* extract granules at 30 mg/kg bw/day for 270 days but there was no significant change after recovery (Table 6). On day 298, it was found that total bilirubin and direct bilirubin were significantly increased in females given* Z. cassumunar* extract granules at 30 mg/kg bw/day (*P* < 0.05). A reduction in total protein was observed in females fed* Z. cassumunar* extract granules at 30 or 1,125 mg/kg bw/day on day 298. SGOT were statistically significantly decreased in female rats fed the test substance at 1,125 mg/kg bw/day on day 298.

Male rats fed with* Z. cassumunar* extract granules at doses of 11.25 or 1,125 mg/kg bw/day for 270 days revealed an increase in BUN. An increase in serum albumin was observed in male rats given* Z. cassumunar* extract granules at doses of 0.3, 30, 11.25, or 112.5 mg/kg bw/day for 270 days compared with the control group. However, there was no significant change in serum albumin in satellite groups. Treatment of male rats with the* Z. cassumunar* extract granules at 3 and 30 mg/kg bw/day for 270 days induced a decrease of blood glucose. A decrease of total protein was observed in male rats fed with* Z. cassumunar* extract granules at 30 mg/kg bw/day on day 298.

#### 3.3.4. Macroscopic and Histological Observations

**Figure 3:**
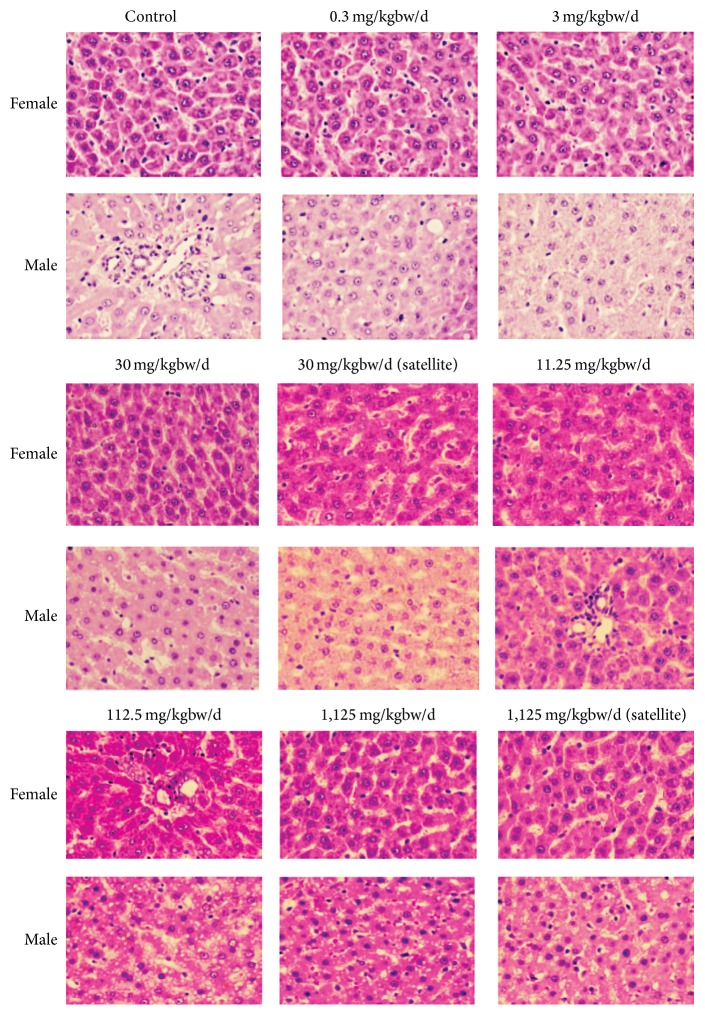
Photomicrographs of liver sections obtained from rats untreated (control) and rats treated with indicated doses of* Z. cassumunar* extract granules in chronic oral toxicity study (hematoxylin and eosin stain, 100x).

**Figure 4:**
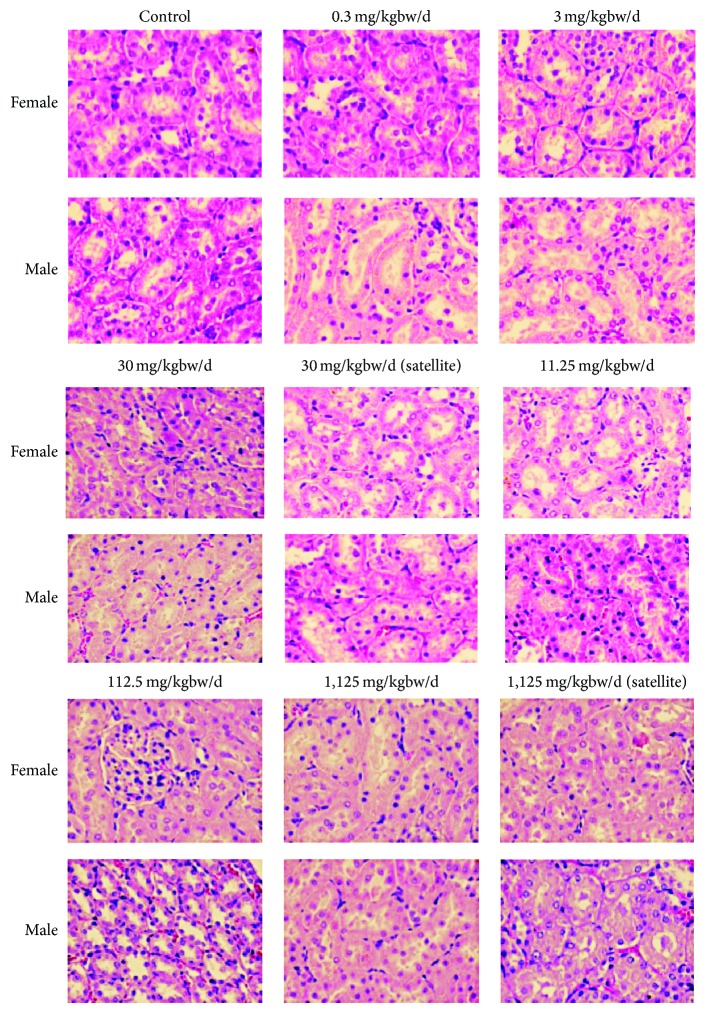
Photomicrographs of kidney sections obtained from rats untreated (control) and rats treated with indicated doses of* Z. cassumunar* extract granules in chronic oral toxicity study (hematoxylin and eosin stain, 100x).

**Figure 5:**
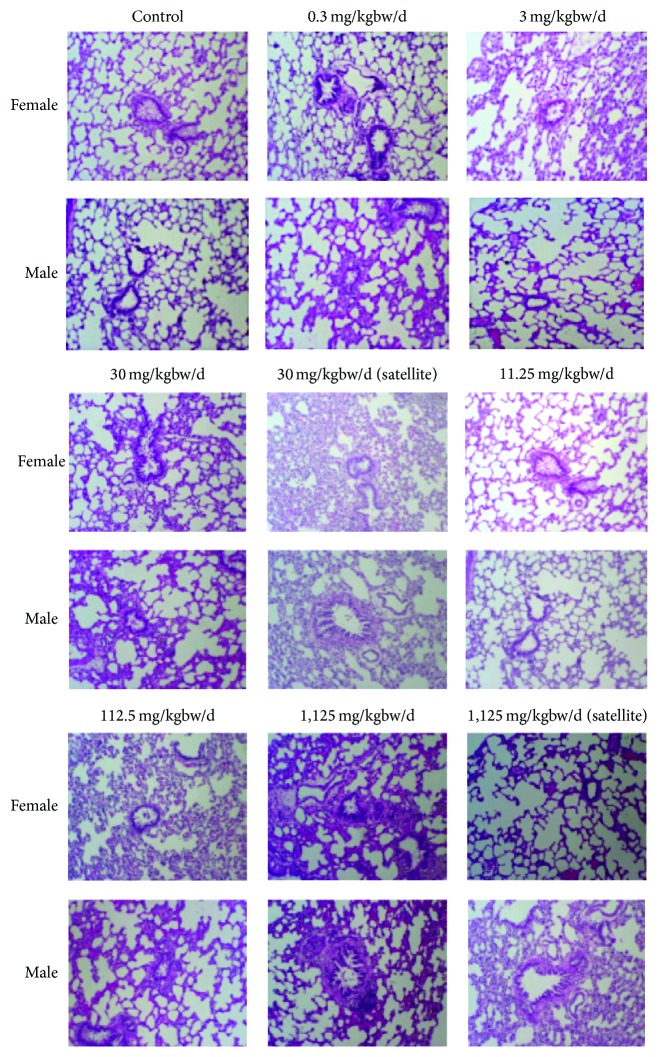
Photomicrographs of lung sections obtained from rats untreated (control) and rats treated with indicated doses of* Z. cassumunar* extract granules in chronic oral toxicity study (hematoxylin and eosin stain, 100x).

All necropsy observations for animals sacrificed at the end of the study were normal. Histological examinations of heart, lungs, livers, pancreas, spleen, adrenal glands, kidneys, stomach, intestine, thymus, muscle, nerve, eyes, brain, ovary, testes, epididymis, and bone showed no pathological appearance. The histological pictures of principal vital organs (livers, kidneys, and lungs) are shown in Figures 3, 4, and 5.

Taken together, body weight changes, relative organ weights, hematological and blood chemistry, and histological data indicate that there is no chronic toxicity in rats fed* Z. cassumunar* extract granules up to 1,125 mg/kg bw for 270 days.

## 4. Discussion

Complementary and alternative medicine, such as herbal remedies, requires thorough safety and efficacy evaluation due to the growing uses all over the world [26]. Although medicinal plants may produce several biological activities in humans, generally very little is known about their toxicity and the same applies for* Z. cassumunar. *Despite preliminary evidence of its therapeutic benefits [10, 27, 28], a few toxicology studies have been performed on* Z. cassumunar* [19].

In the acute toxicity assessment of this study, male and female rats were fed with* Z. cassumunar* extract granules at single dose of 5,000 mg/kg bw. No mortality or morbidity was observed in any of animals used throughout 14-day observation period. Treated animals did not show any sign of aggression or unusual behavior during handling. Furthermore, clinical observations did not indicate evidence of substance-related toxicity. Therefore the oral LD_50_ value of* Z. cassumunar* extract granules for male and female rats must be greater than 5,000 mg/kg bw.

Measurements of the body weight over the entire period of 14 days found no difference among female groups. In contrast, the treated male rats showed a decrease of body weight gain in comparison with the control group. Generally, a reduction in body weight gain and the weights of internal organs is a simple and sensitive index of toxicity after exposure to a toxic substance [29, 30]. A decrease in body weight gain would be an indicator of adverse effects. Although the weights of certain internal organs of animals treated with* Z. cassumunar* extract granules were statistically different from that in the controls, they did not exhibit any gross morphological changes. Therefore, it appears that* Z. cassumunar* extract is virtually nontoxic.

Our findings are consistent with the results of other researchers [18–20] where LD_50_ of the* Z. cassumunar* rhizome extract was in the range of 10–20 g/kg bw of animals. Altogether, these observations show an absence of acute toxicity of* Z. cassumunar* extract granules at 5000 mg/kg bw. According to the OECD guideline [24] and referring to Kennedy Jr. et al. [31], orally administered* Z. cassumunar *rhizomes could be considered practically nontoxic.

To provide further assurance of safety for* Z. cassumunar*, repeated dose toxicity studies were conducted to evaluate the adverse effects of this plant after prolonged use. Chronic toxicity studies are carried out to provide information about the possible health hazards likely to arise from repeated exposure over a relatively limited period of time including information about target organs, the possibilities of cumulative effects, and an estimate of the dose at which there is no observed adverse effect. Feeding* Z. cassumunar* extract granules at doses of 0.3, 3, 30, 11.25, 112.5, and 1,125 mg/kg bw/day for 270 consecutive days did not produce any clinical signs of toxicity and death in experimental rats. It should be noted that the doses of* Z. cassumunar *extract granules employed in the present study represent up to 10 times more than those used as an antiasthmatic agent [10, 11].

We found that no significant difference in body weight was observed between experimental and control animals. Our findings were not consistent with a previous study [20] which demonstrated the toxicity of* Z. cassumunar *in rats. The discrepancy of the results may be explained by difference in experimental designs. In the previous paper, the authors either daily force-fed Wistar rats or allowed them to eat food mixed with* Z. cassumunar *powder naturally (*ad libitum*) for 12 months. They reported a decrease in food intake and body weight gain in male rats, compared to the control. A reduction in food intake may be due to the unpalatability of the crude* Z. cassumunar* powder. This may cause a decrease in body weights of treated male rats. It is also possible that the inconsistent results may be due to different rat strains used and possibly from the prolonged force-feeding.

Blood is an important index of physiological and pathological status in man and animals. The parameters usually measured are red blood cell, white blood cells, differentiated leukocyte count, platelet, hematocrit, and hemoglobin content [32]. The hematopoietic system is very sensitive to toxic compounds. The normal range of these hematological parameters can be altered by the ingestion of some toxic plants. Our study showed that 270-day feeding of* Z. cassumunar* extract granules in the dose range of 0.3−1,125 mg/kg bw/day did not show treatment-related biologically significant adverse effect of the most hematological parameters and blood chemistry indices in rats. Although some hematological parameters showed statistical differences compared to the control groups these differences were minor and not dose related. This may be the result of biological variations among rats rather than the treatment effects. In addition, all values of parameter obtained through that period were still within the normal range [33].

Kidney is a sensitive organ, and its functions are affected by a number of factors including drugs and phytochemicals of plant origin [34, 35]. In our study, serum urea and creatinine levels were used to assess the possible renal damage due to* Z. cassumunar.* Male rats fed with* Z. cassumunar *extract granules at the doses of 11.25 or 1,125 mg/kg bw/day for 270 days revealed an increase in BUN. However, the values are still within the reference intervals for Sprague-Dawley rats [33], and feeding rats with the test substance did not change the creatinine levels. In addition, the normal appearance of kidneys in experimental animals confirmed that there is no toxic effect of* Z. cassumunar *on kidneys. Therefore, changes in the BUN values in the groups treated with* Z. cassumunar* were not considered to associate with toxicity.

Hepatocytes are especially liable to injury because of their functions of taking up and dealing with many metabolites, drugs, and other toxic substances. The present study shows no significant increase in liver weights of the treated groups when compared with the control. Histological examinations of the liver show normal histological appearance in the treated groups as also seen in the control group. Additionally, the normal histology observed in the liver is in agreement with normal levels of liver function tests. Therefore, our findings suggest that the* Z. cassumunar *extracts used in the study are not hepatotoxic.

Our findings in chronic toxicity assessment are contrary to a previous study [20], which reported liver abnormality in certain groups of Wistar rats and* Macaca fascicularis* monkeys, fed crude* Z. cassumunar* powder. The authors also demonstrated that high fluctuation of SGOT and SGPT levels were observed in some treated animals. However, it should be noted that, in their studies, liver cirrhosis and carcinogenesis occurred due to dose irregularity. Therefore, in the light of the present study, it is possible that hepatotoxicity reported in the previous study may be due to other factors and had no connection with the chronic toxicity of* Z. cassumunar*. It may be concluded that the results of chronic toxicity study presented here are a further confirmation of the safety of* Z. cassumunar* rhizomes. Our findings were similar to the findings of previous studies [18, 19] justifying its traditional claim in the treatment of various diseases. However, this claim demands for further clinical studies.

## 5. Conclusion

In conclusion, as demonstrated by the findings of 14-day acute oral toxicity and 270-day chronic repeated oral doses with 28-day recovery phase,* Z. cassumunar* extract granules were well tolerated in the rat and did not induce any toxic effect. The oral no-observed-adverse-effect level (NOAEL) for* Z. cassumunar* extract granules was 1,125 mg/kg bw/day, the highest doses tested in male and female rats.

## Figures and Tables

**Table 1:** Body weights (g) of rats receiving a single dose of *Z*. *cassumunar* extract granules in acute toxicity study^a^.

Dose (mg/kg bw/day)	Day 0	Day 7	Day 14	Weight gain at day 14
Female				
0	215.00 ± 2.69	237.00 ± 4.78	231.00 ± 3.86	16.00 ± 3.86
5,000	215.50 ± 3.05	227.80 ± 5.28	233.30 ± 4.56	17.80 ± 2.37
Male				
0	240.00 ± 3.33	291.70 ± 4.49	341.70 ± 4.08	101.70 ± 2.20
5,000	242.20 ± 2.78	275.00 ± 3.82	307.20 ± 5.66^*^	65.00 ± 3.53^*^

^a^Values are expressed as mean ± SEM (*n* = 10 for each group).

^*^ *P* values < 0.05, compared to the corresponding control.

**Table 2:** Absolute organ weights (g) of rats receiving a single dose of *Z*. *cassumunar* extract granules for 14 days^a^.

Organ	Control	5000 mg/kg bw
Female		
Brain	1.85 ± 0.02	1.82 ± 0.03
Lungs	1.10 ± 0.03	1.27 ± 0.05^*^
Heart	0.85 ± 0.02	0.81 ± 0.02
Livers	6.61 ± 0.31	7.08 ± 0.30
Spleen	0.72 ± 0.02	0.85 ± 0.06
Pancreas	0.58 ± 0.02	0.55 ± 0.04
Adrenals	0.03 ± 0.00	0.03 ± 0.00
Kidneys	0.78 ± 0.01	0.84 ± 0.02^*^
Ovary	0.06 ± 0.00	0.06 ± 0.00
Uterus	0.49 ± 0.03	0.50 ± 0.03
Male		
Brain	1.99 ± 0.01	1.95 ± 0.02
Lungs	1.38 ± 0.03	1.32 ± 0.05
Heart	1.10 ± 0.02	1.07 ± 0.03
Livers	10.36 ± 0.21	11.13 ± 0.35
Spleen	1.00 ± 0.03	0.88 ± 0.04^*^
Pancreas	0.69 ± 0.03	0.73 ± 0.03
Adrenals	0.03 ± 0.00	0.03 ± 0.00
Kidneys	1.25 ± 0.03	1.18 ± 0.02
Testes	1.72 ± 0.02	1.70 ± 0.01
Epididymis	0.54 ± 0.01	0.49 ± 0.01^*^

^a^Values are expressed as mean ± SEM (*n* = 10 for each group).

^*^ *P* values < 0.05, compared to the corresponding control.

**Table 3:** Body weights (g) of rats treated with *Z*. *cassumunar* extract granules in chronic oral toxicity study^a^.

Dose (mg/kg bw/d)	Day 0	Day 90	Day 180	Day 270	Day 298	Weight gain at day 270
Female						
0	220.50 ± 4.10	312.5 ± 6.93	357.50 ± 9.50	392.00 ± 3.75		171.50 ± 2.71
0.3	218.50 ± 1.38	302.50 ± 4.03	353.50 ± 7.27	386.50 ± 5.74		168.00 ± 4.57
3.0	229.5 0 ± 2.29	302.00 ± 7.04	353.50 ± 11.76	388.00 ± 4.85		158.50 ± 5.08
30	221.50 ± 4.83	310.00 ± 5.47	350.00 ± 4.64	385.50 ± 7.14		164.00 ± 2.64
30^b^	218.00 ± 7.67	310.00 ± 7.67	349.00 ± 7.67	383.50 ± 3.97	400.50 ± 4.73	165.50 ± 7.03
11.25	226.50 ± 3.02	307.50 ± 5.19	353.00 ± 7.67	391.00 ± 3.82		164.50 ± 2.14
112.5	225.50 ± 11.76	305.50 ± 11.76	353.50 ± 11.76	389.50 ± 4.25		164.00 ± 3.45
1,125	225.00 ± 4.64	317.00 ± 4.64	357.00 ± 4.64	387.00 ± 5.04		162.00 ± 3.02
1,125^b^	217.00 ± 7.67	308.00 ± 7.67	356.00 ± 7.67	386.50 ± 2.94	403.50 ± 5.31	169.50 ± 4.62
Male						
0	261.00 ± 4.10	529.00 ± 7.81	605.50 ± 6.93	685.50 ± 5.63		424.50 ± 2.10
0.3	258.50 ± 1.38	506.00 ± 5.64	580.50 ± 4.03	640.50 ± 2.53		382.00 ± 1.98
3.0	253.50 ± 2.29	521.50 ± 4.89	600.00 ± 7.04	675.00 ± 5.14		421.50 ± 3.19
30	253.50 ± 4.83	524.00 ± 4.42	599.00 ± 5.47	676.50 ± 3.67		423.00 ± 3.73
30^b^	248.50 ± 3.02	544.50 ± 4.47	607.50 ± 5.19	677.50 ± 3.49	697.50 ± 4.01	429.00 ± 2.12
11.25	262.50 ± 3.02	529.50 ± 4.47	602.50 ± 5.19	682.50 ± 4.29		420.00 ± 1.52
112.5	266.00 ± 3.05	533.00 ± 6.86	616.50 ± 5.64	676.50 ± 4.34		410.50 ± 1.35
1,125	268.50 ± 3.69	523.50 ± 4.62	592.50 ± 6.20	682.50 ± 3.20		414.00 ± 1.96
1,125^b^	250.00 ± 4.36	519.00 ± 9.95	597.00 ± 11.62	677.00 ± 5.82	698.50 ± 3.24	427.00 ± 3.46

^a^Values are expressed as mean ± SEM (*n* = 10 for each group).

^b^Satellite groups were treated with *Z*. *cassumunar* extract granules at the indicated dose for 270 days followed by no treatment for 28 days.

**Table 4:** Absolute organ weights (g) of rats treated with *Z*. *cassumunar* extract granules in chronic oral toxicity study^a^.

Organ	Dose (mg/kg bw/d)								
	0	0.3	3.0	30	30^b^	11.25	112.5	1,125	1,125^b^
Female									
Brain	1.94 ± 0.02	1.93 ± 0.03	1.98 ± 0.03	1.91 ± 0.03	1.90 ± 0.02	1.96 ± 0.03	1.93 ± 0.02	1.90 ± 0.02	1.90 ± 0.02
Lungs	2.18 ± 0.14	2.16 ± 0.15	2.43 ± 0.24	2.08 ± 0.15	2.00 ± 0.22	2.31 ± 0.27	2.29 ± 0.14	2.35 ± 0.26	2.30 ± 0.31
Heart	1.24 ± 0.03	1.31 ± 0.03	1.30 ± 0.03	1.29 ± 0.03	1.35 ± 0.06	1.31 ± 0.03	1.46 ± 0.05^*^	1.37 ± 0.03^*^	1.21 ± 0.04
Livers	9.12 ± 0.88	10.12 ± 0.21	9.84 ± 0.46	10.06 ± 0.23	10.17 ± 0.24	10.32 ± 0.31	9.77 ± 0.37	9.42 ± 0.29	9.38 ± 0.29
Spleen	0.81 ± 0.04	0.81 ± 0.04	0.84 ± 0.04	0.78 ± 0.05	0.76 ± 0.02	0.73 ± 0.04	0.70 ± 0.01^*^	0.74 ± 0.04	0.72 ± 0.03^*^
Adrenals	0.04 ± 0.00	0.06 ± 0.02	0.04 ± 0.00	0.04 ± 0.00	0.03 ± 0.00	0.04 ± 0.00	0.04 ± 0.00	0.04 ± 0.00	0.03 ± 0.00
Kidneys	1.32 ± 0.02	1.29 ± 0.02	1.25 ± 0.03^*^	1.25 ± 0.02^*^	1.24 ± 0.02^*^	1.27 ± 0.02	1.29 ± 0.02	1.28 ± 0.02	1.20 ± 0.02^*^
Ovary	0.06 ± 0.00	0.06 ± 0.00	0.06 ± 0.01	0.07 ± 0.00	0.06 ± 0.01	0.06 ± 0.00	0.06 ± 0.02	0.06 ± 0.00	0.05 ± 0.00
Uterus	1.36 ± 0.31	1.34 ± 0.17	1.51 ± 0.34	2.71 ± 0.53	2.31 ± 0.81	1.19 ± 0.07	1.17 ± 0.13	1.45 ± 0.29	1.33 ± 0.12
Male									
Brain	2.04 ± 0.05	2.03 ± 0.03	2.05 ± 0.04	2.04 ± 0.03	2.12 ± 0.02	2.06 ± 0.02	1.98 ± 0.05	2.05 ± 0.02	2.04 ± 0.02
Lung	2.74 ± 0.31	2.79 ± 0.19	2.73 ± 0.22	3.52 ± 0.36^*^	2.70 ± 0.13	2.63 ± 0.27	2.79 ± 0.14	2.75 ± 0.26	2.63 ± 0.31
Heart	1.78 ± 0.03	1.70 ± 0.04	1.82 ± 0.06	1.96 ± 0.08^*^	2.09 ± 0.04^*^	1.71 ± 0.03	1.73 ± 0.05	1.77 ± 0.03	1.81 ± 0.04
Livers	17.79 ± 0.55	17.02 ± 0.92	16.77 ± 0.55	17.38 ± 1.11	18.57 ± 0.76	17.32 ± 0.31	16.77 ± 0.37	17.42 ± 0.29	17.38 ± 0.29
Spleen	1.01 ± 0.03	1.02 ± 0.04	1.00 ± 0.04	1.02 ± 0.06	1.05 ± 0.03	1.03 ± 0.04	1.07 ± 0.01^*^	1.04 ± 0.04	1.02 ± 0.03
Adrenals	0.03 ± 0.02	0.03 ± 0.00	0.03 ± 0.00	0.03 ± 0.00	0.04 ± 0.00	0.04 ± 0.00	0.04 ± 0.00	0.04 ± 0.00	0.03 ± 0.00
Kidneys	1.78 ± 0.05	1.88 ± 0.05	1.92 ± 0.06^*^	1.87 ± 0.06	2.05 ± 0.04^*^	1.77 ± 0.02	1.79 ± 0.02	1.88 ± 0.02	1.82 ± 0.02
Testes	2.01 ± 0.04	2.04 ± 0.02	2.02 ± 0.03	2.07 ± 0.05	2.08 ± 0.03	2.06 ± 0.03	2.08 ± 0.05	2.06 ± 0.04	2.05 ± 0.06
Epididymis	0.88 ± 0.03	0.85 ± 0.01	0.85 ± 0.02	0.83 ± 0.03	0.84 ± 0.01	0.94 ± 0.07	0.87 ± 0.03	0.95 ± 0.09	0.83 ± 0.02

^a^Values are expressed as mean ± SEM (*n* = 10 for each group).

^b^Satellite groups were treated with *Z*. *cassumunar* extract at the indicated dose for 270 days followed by no treatment for 28 days.

^*^ *P* values < 0.05, compared to the corresponding control.

**Table 5:** Hematological parameters of rats treated with *Z*. *cassumunar* extract granules in chronic oral toxicity study^a^.

Parameter	Dose (mg/kg bw/d)								
	0	0.3	3.0	30	30^b^	11.25	112.5	1,125	1,125^b^
Female									
WBC (×10^3^/*μ*L)	2.14 ± 0.26	2.45 ± 0.34	2.02 ± 0.26	2.43 ± 0.28	1.80 ± 0.21	2.17 ± 0.14	2.44 ± 0.17	2.44 ± 0.22	2.76 ± 0.35
RBC (×10^6^/*μ*L)	7.33 ± 0.09	7.23 ± 0.13	7.35 ± 0.12	7.33 ± 0.17	7.35 ± 0.12	7.23 ± 0.26	7.43 ± 0.16	7.37 ± 0.15	7.36 ± 0.26
HGB (g/dL)	14.80 ± 0.13	14.74 ± 0.25	14.63 ± 0.19	14.99 ± 0.28	14.88 ± 0.21	15.80 ± 0.35	15.90 ± 0.23	15.15 ± 0.21	15.78 ± 0.44
HCT (%)	44.60 ± 0.52	43.70 ± 0.76	43.80 ± 0.68	43.70 ± 1.14	43.60 ± 0.54	41.90 ± 1.46	41.80 ± 0.87	44.60 ± 0.60	45.10 ± 1.52
MCV (fL)	60.60 ± 0.25	59.98 ± 0.34	59.48 ± 0.38^*^	59.73 ± 0.56^*^	59.27 ± 0.31^*^	59.30 ± 0.21	59.60 ± 0.31	58.00 ± 0.33	59.20 ± 0.36
MCH (pg)	20.07 ± 0.15	20.39 ± 0.19	19.91 ± 0.12	20.47 ± 0.26	20.26 ± 0.16	17.51 ± 0.22^*^	19.72 ± 0.13	18.75 ± 0.13	18.04 ± 0.15
MCHC (g/dL)	33.13 ± 0.21	34.01 ± 0.32^*^	33.49 ± 0.23	34.30 ± 0.49^*^	34.18 ± 0.26∗	30.51 ± 0.35^*^	33.73 ± 0.11	34.65 ± 0.17	33.60 ± 0.24
PLT (×10^5^/*μ*L)	6.77 ± 0.30	7.19 ± 0.25	6.64 ± 0.34	7.22 ± 0.52	6.62 ± 0.14	6.76 ± 0.03	6.81 ± 0.02	6.73 ± 0.02	6.71 ± 0.04
Differential count of WBC (%)									
NEUT	22.80 ± 3.11	17.70 ± 3.29	18.90 ± 1.38	18.90 ± 1.53	24.50 ± 2.68	21.78 ± 0.15	21.73 ± 0.94	21.09 ± 0.10	22.07 ± 0.20
LYMPH	67.90 ± 3.08	70.90 ± 3.47	73.10 ± 1.65	74.70 ± 2.26	63.60 ± 2.49	70.28 ± 0.19	64.56 ± 0.29	63.25 ± 0.25	66.61 ± 0.23
MONO	7.00 ± 0.42	8.20 ± 0.53	8.20 ± 0.25	7.60 ± 0.65	7.40 ± 0.62	7.32 ± 0.22	7.12 ± 0.04	7.10 ± 0.03	7.08 ± 0.01
EO	2.30 ± 0.45	2.20 ± 0.65	2.80 ± 0.49	3.80 ± 0.63	2.50 ± 1.22	2.42 ± 0.00	2.03 ± 0.02	2.01 ± 0.01	2.02 ± 0.01
BASO	0.00 ± 0.00	0.00 ± 0.00	0.00 ± 0.00	0.00 ± 0.00	0.00 ± 0.00	0.00 ± 0.00	0.00 ± 0.00	0.00 ± 0.00	0.00 ± 0.00

Male									
WBC (×10^3^/*μ*L)	3.06 ± 0.29	3.24 ± 0.28	3.34 ± 0.23	3.44 ± 0.24	3.06 ± 0.30	3.54 ± 0.44	3.29 ± 0.34	3.09 ± 0.27	3.86 ± 0.56
RBC (×10^6^/*μ*L)	8.07 ± 0.11	8.04 ± 0.16	8.41 ± 0.12^*^	8.16 ± 0.11	8.45 ± 0.15^*^	8.04 ± 0.11	8.17 ± 0.63	8.09 ± 0.16	8.03 ± 0.11
HGB (g/dL)	15.51 ± 0.13	15.56 ± 0.16	15.92 ± 0.18	15.93 ± 0.21	15.85 ± 0.17	15.84 ± 0.20	14.62 ± 1.04	15.42 ± 0.19	15.66 ± 0.12
HCT (%)	47.10 ± 0.43	47.40 ± 0.70	48.40 ± 0.62	47.70 ± 0.52	47.80 ± 0.83	51.10 ± 0.72^*^	47.60 ± 3.72	47.90 ± 0.77	51.00 ± 0.54^*^
MCV (fL)	58.44 ± 0.48	59.03 ± 0.54	57.55 ± 0.35	58.41 ± 0.49	56.47 ± 0.18^*^	59.10 ± 0.31	59.30 ± 0.42	58.90 ± 0.50	57.30 ± 0.37
MCH (pg)	19.25 ± 0.19	19.40 ± 0.32	18.92 ± 0.12	19.52 ± 0.17	18.81 ± 0.35	18.35 ± 0.18	18.03 ± 0.30	18.18 ± 0.18	17.62 ± 0.11
MCHC (g/dL)	32.94 ± 0.19	32.85 ± 0.28	32.90 ± 0.15	33.43 ± 0.24	33.29 ± 0.59	31.97 ± 0.26	32.43 ± 0.54	31.89 ± 0.22	32.83 ± 0.15
PLT (×10^5^/*μ*L)	7.91 ± 0.16	8.19 ± 0.24	7.89 ± 0.23	7.90 ± 0.15	7.36 ± 0.21	8.09 ± 0.24	7.89 ± 0.23	7.90 ± 0.15	7.36 ± 0.21
Differential count of WBC (%)									
NEUT	12.30 ± 1.22	13.00 ± 2.59	14.00 ± 1.84	15.80 ± 1.11	12.50 ± 1.78	12.22 ± 0.34	12.25 ± 0.23	12.45 ± 0.22	12.70 ± 0.27
LYMPH	72.10 ± 1.81	69.50 ± 1.43	69.80 ± 1.91	70.10 ± 0.75	74.80 ± 2.88	73.83 ± 0.29	73.88 ± 0.34	73.42 ± 0.27	74.02 ± 0.47
MONO	10.80 ± 0.93	9.50 ± 1.19	9.20 ± 0.44	10.60 ± 0.96	10.40 ± 0.72	10.09 ± 0.02	10.15 ± 0.05	10.20 ± 0.04	10.13 ± 0.04
EO	4.80 ± 0.53	3.90 ± 0.42^*^	4.00 ± 0.45	3.80 ± 0.54	4.30 ± 0.92	4.00 ± 0.00	4.02 ± 0.01	5.02 ± 0.01	5.01 ± 0.01
BASO	0.00 ± 0.00	0.00 ± 0.00	0.00 ± 0.00	0.00 ± 0.00	0.00 ± 0.00	0.00 ± 0.00	0.00 ± 0.00	0.00 ± 0.00	0.00 ± 0.00

^a^Values are expressed as mean ± SEM (*n* = 10 for each group).

^b^Satellite groups were treated with *Z*. *cassumunar* extract granules at the indicated dose for 270 days followed by no treatment for 28 days.

^*^ *P* values < 0.05, compared to the corresponding control.

WBC, white blood cell count; RBC, red blood cell count; HGB, hemoglobin concentration; HCT, hematocrit; MCV, mean corpuscular volume; MCH, mean corpuscular hemoglobin; MCHC, mean corpuscular hemoglobin concentration; PLT, platelet count; NEUT, neutrophil; LYMPH, lymphocyte; MONO, monocyte; EO, eosinophil; BASO, basophil.

**Table 6:** Clinical chemistry parameters of rats treated with *Z. cassumunar* extract granules in chronic oral toxicity study^a^.

Parameter	Dose (mg/kg bw/d)								
	0	0.3	3.0	30	30^b^	11.25	112.5	1,125	1,125^b^
Female									
GLU (mg/dL)	127.90 ± 3.55	131.60 ± 4.00	131.60 ± 3.43	128.30 ± 4.26	133.90 ± 4.65	124.90 ± 7.80	125.60 ± 18.45	124.00 ± 4.36	129.80 ± 10.24
BUN (mg/dL)	21.50 ± 0.87	24.00 ± 0.79^*^	23.40 ± 0.70	23.00 ± 0.83	21.60 ± 0.65	21.93 ± 0.35	25.17 ± 3.27^*^	23.00 ± 0.68	20.14 ± 1.07
CRE (mg/dL)	0.45 ± 0.02	0.47 ± 0.03	0.42 ± 0.02	0.43 ± 0.02	0.41 ± 0.01	0.43 ± 0.02	0.44 ± 0.02	0.42 ± 0.02	0.46 ± 0.01
TP (g/dL)	6.21 ± 0.10	6.10 ± 0.10	6.28 ± 0.17	6.02 ± 0.17	5.88 ± 0.08^*^	6.10 ± 0.10	6.28 ± 0.17	6.02 ± 0.17	5.68 ± 0.08^*^
ALB (g/dL)	4.31 ± 0.07	4.19 ± 0.07	4.23 ± 0.14	4.09 ± 0.13	3.88 ± 0.06^*^	4.29 ± 0.00	4.18 ± 0.01^*^	4.39 ± 0.00	4.16 ± 0.02^*^
T-BIL (mg/dL)	0.26 ± 0.02	0.24 ± 0.02	0.23 ± 0.01	0.23 ± 0.01	0.29 ± 0.01	0.13 ± 0.02	0.15 ± 0.02	0.11 ± 0.02	0.10 ± 0.01
D-BIL (mg/dL)	0.01 ± 0.01	0.02 ± 0.01	0.00 ± 0.00	0.01 ± 0.01	0.01 ± 0.01	0.13 ± 0.02	0.15 ± 0.02	0.11 ± 0.02	0.10 ± 0.01
SGOT (U/L)	172.20 ± 10.51	229.90 ± 29.41^*^	156.60 ± 11.94	223.40 ± 21.15^*^	184.20 ± 10.86	181.80 ± 24.26	185.20 ± 17.43	162.70 ± 18.24	240.90 ± 33.25^*^
SGPT (U/L)	51.50 ± 5.28	56.60 ± 12.65	51.30 ± 3.88	50.30 ± 21.76	53.70 ± 4.90	52.20 ± 5.50	55.60 ± 6.32	52.20 ± 6.77	77.90 ± 10.29^*^
ALP (U/L)	34.20 ± 1.57	31.60 ± 1.85	31.30 ± 1.64	30.00 ± 2.19	33.30 ± 1.68	27.20 ± 2.41^*^	33.30 ± 3.62	34.80 ± 1.52	31.40 ± 3.79
Male									
GLU (mg/dL)	135.20 ± 2.46	131.50 ± 2.76	138.30 ± 3.36	131.20 ± 3.16	136.00 ± 3.34	125.00 ± 1.76	126.10 ± 3.61	129.30 ± 4.62	124.10 ± 4.90
BUN (mg/dL)	20.20 ± 0.51	21.90 ± 1.46	21.90 ± 0.74	21.40 ± 1.05	20.70 ± 0.80	22.00 ± 0.80	19.90 ± 0.62	20.40 ± 0.62	19.10 ± 0.82
CRE (mg/dL)	0.40 ± 0.02	0.40 ± 0.02	0.41 ± 0.03	0.42 ± 0.02	0.40 ± 0.03	0.40 ± 0.02	0.36 ± 0.02	0.36 ± 0.02	0.36 ± 0.02
TP (g/dL)	6.03 ± 0.13	6.17 ± 0.08	6.19 ± 0.05	6.15 ± 0.10	5.72 ± 0.08^*^	6.36 ± 0.06	6.54 ± 0.07	6.39 ± 0.09	6.54 ± 0.14
ALB (g/dL)	3.70 ± 0.07	3.71 ± 0.06	3.75 ± 0.03	3.74 ± 0.07	3.61 ± 0.06	3.68 ± 0.05	3.75 ± 0.04	3.70 ± 0.04	3.92 ± 0.07
T-BIL (mg/dL)	0.21 ± 0.02	0.19 ± 0.01	0.20 ± 0.00	0.18 ± 0.01	0.23 ± 0.01	0.23 ± 0.02	0.22 ± 0.01	0.23 ± 0.02	0.21 ± 0.01
D-BIL (mg/dL)	0.02 ± 0.01	0.00 ± 0.00	0.01 ± 0.01	0.00 ± 0.00	0.01 ± 0.01	0.00 ± 0.0	0.00 ± 0.00	0.01 ± 0.01	0.00 ± 0.00
SGOT (U/L)	174.80 ± 14.62	191.90 ± 18.66	183.20 ± 13.41	206.00 ± 27.19	157.20 ± 26.97	179.90 ± 7.20	173.40 ± 14.34	175.60 ± 10.38	194.10 ± 22.75
SGPT (U/L)	56.60 ± 4.08	50.40 ± 2.62	57.90 ± 3.67	62.80 ± 9.13	69.10 ± 15.58	52.00 ± 4.27	53.90 ± 5.51	54.20 ± 4.39	66.40 ± 8.71
ALP (U/L)	49.40 ± 2.27	55.00 ± 2.26	55.80 ± 2.17	55.10 ± 1.70	50.50 ± 3.66	43.50 ± 1.58	53.40 ± 2.94	50.10 ± 1.68	49.70 ± 1.93

^a^Values are expressed as mean ± SEM (*n* = 10 for each group).

^b^Satellite groups were treated with *Z*. *cassumunar* extract granules at the indicated dose for 270 days followed by no treatment for 28 days.

^*^ *P* values < 0.05, compared to the corresponding control.

GLU, glucose; BUN, blood urea nitrogen; CRE, creatinine; TP, total protein; ALB, albumin; T-BIL, total bilirubin; D-BIL, direct bilirubin; SGOT, serum glutamic oxaloacetic transaminase; SGPT, serum glutamine-pyruvic transaminase; ALP, alkaline phosphatase.
